# Femoral derotation osteotomy with multi-level soft tissue procedures in children with cerebral palsy: Does it improve gait quality?

**DOI:** 10.1007/s11832-015-0706-4

**Published:** 2015-11-23

**Authors:** Yavuz Saglam, N. Ekin Akalan, Yener Temelli, Shavkat Kuchimov

**Affiliations:** Orthopedics and Traumatology Department, Bahcelievler State Hospital, Istanbul, Turkey; Department of Physiotherapy and Rehabilitation, Faculty of Health Science, Istanbul University, Istanbul, Turkey; Motion Analysis Laboratory, Istanbul, Turkey; Orthopedics and Traumatology Department, Istanbul Faculty of Medicine, Istanbul University, Istanbul, Turkey; Institute of Biomedical Engineering, Bogazici University, Istanbul, Turkey

**Keywords:** Femoral anteversion assessment, Cerebral palsy, Femoral derotational osteotomy, Gait analysis

## Abstract

**Purpose:**

Poor motor control and delayed thumb function and a delay in walking are the main factors which retard the natural decrease of the femoral anteversion (FA) with age. In addition, cerebral palsy (CP) patients usually have muscular imbalance around the hip as well as muscle contractures, both of which are main factors accounting for the increased FA which is commonly present in CP patients. The purpose of this retrospective study was to analyze the mid-term results of femoral derotational osteotomy (FDO) on the clinical findings, temporospatial and kinematic parameters of gait in children with CP.

**Methods:**

We performed a retrospective review of all patients diagnosed with CP and increased FA who were treated with FDO with multi-level soft tissue surgeries at a single institution between 1992 and 2011. FA assessment was done in the prone position, and internal (IR) and external rotation (ER) of the hip was measured in the absence of pelvis rotation. Surgical procedures were performed on the basis of both clinical findings and video analysis. Clinical findings, Edinburgh Visual Gait Scores (EVGS) and results from three-dimensional gait analysis were analyzed preoperatively and last follow-up.

**Results:**

A total of 93 patients with 175 affected extremities were included in this review. Mean age was 6.2 ± 3.1 (standard deviation) at initial surgery. The average length of the follow-up period was 6.3 ± 3.7 years. At the last follow-up, the postoperative hip IR had significantly decreased (73.9° vs. 46.2°; *p* < 0.0001), the hip ER had significantly improved (23.8° vs. 37°; *p* < 0.0001) and the popliteal angle had significantly decreased (64.2° vs. 55.8°; *p* < 0.0001). The total EVGS showed significant improvement after FDO (35.2 ± 6.4 vs. 22.5 ± 6.1; *p* < 0.001). Computed gait analysis showed significant improvement in the foot progression angle (FPA; 8.1° vs. −16.9°; *p* = 0.005) and hip rotation (−13.9° vs. 5.7°; *p* = 0.01) at the last follow-up. Stance time was improved (60.2 vs. 65.1 %; *p* = 0.02) and swing time was decreased (39.9 vs. 35.2 %; *p* = 0.03). Double support time and cadence were both decreased (*p* = 0.032 and *p* = 0.01).

**Conclusions:**

Our data suggest that the FDO is an appropriate treatment strategy for the correction of FA and associated in-toeing gait in children with CP. Improvements in clinical and kinematic parameters were observed in both groups after FDO with multi-level soft tissue release. The most prominent effects of FDO were on transverse plane hip rotation and FPA.

## Introduction

Cerebral palsy (CP) is a disorder which primarily affects body movement and muscle coordination. It is characterized by spasticity and progressive musculo-skeletal problems [1–3]. The management of spasticity is a major challenge and initially focuses on the elimination of spasticity at an early age [4–6]. Uncontrolled spasticity may gradually cause progressive muscle contractures and bone deformities, such as increased femoral anteversion (FA) [7]. The primary main aim of treating a CP patient is to create a productive, highly functional and active individual in his/her social environment [8, 9].

The physiological high anteversion present at birth (40–60°) slowly decreases with growth in normally developing children. However, in children with CP, the normal remodeling process of the FA does not occur [10], and FA has not been found to resolve over time in CP patients [11]. Poor motor control and delayed functional milestones, such as delays in rolling-over, crawling, kneeling, standing and walking, are the main factors which retard the natural decrease of the FA with age. In addition, CP patients usually have muscular imbalance around the hip as well as muscle contractures, both of which are main factors accounting for the increased FA which is commonly present in CP patients [8]. A growing child with CP usually has internal rotation contracture of the hip, which also contributes to a decrease of the FA angle [12]. In children with CP, torsion occurs as a gentle twisting throughout the whole femur [10].

Many clinical and radiological methods have been developed to diagnose increased FA [13–19]. In the clinical setting, goniometric measurement of hip internal–-external rotation, the prominence angle test and, for more precise measurements, computed tomography (CT) have been used to determine the existence of increased FA. Even though CT-based methods have been reported to deliver the most accurate measurement, physical examination is a frequently performed, cost-effective, and safe method that does not involve exposure of the child to radiation [14, 20]. Femoral derotational osteotomies (FDO) are being increasingly performed, and there have been many reports in support of this approach to resolve this mal-torsion [8, 18, 21–23]. This is a key strategy for treating CP patients and the primary procedure adopted to improve the in-toeing gait in the transverse plane. This procedure also has a number of effects on the coronal and sagittal planes of the gait [11]. The method of de-rotation is still controversial, with each de-rotation technique associated with specific benefits and problems [22, 24].

The aim of this retrospective study was to analyze the mid-term results of FDO on the clinical findings and the temporospatial and kinematic parameters of gait in children with CP.

## Methods

This study was a retrospective review of all patients diagnosed with CP and increased FA who were treated with FDO with multi-level soft tissue surgeries at a single institution between 1992 and 2011. The inclusion criteria used for patient selection were: medical history for >1 year of follow-up; spastic diplegia and tetraplegia; data available on the pre- and post-operative clinical examination; Gross Motor Function Classification System level I, II or III [25]; spastic type CP [1]. The medical charts of all patients meeting these criteria were analyzed for demographic data, clinical findings (hip flexion and extension, hip flexion contracture (Thomas test), hip abduction angle, femur internal and external rotations, total rotation arc of the hip, popliteal angle, knee flexion contracture, thigh–foot angle, rectus contracture (Duncan Ely test) and motion analysis parameters. All clinical findings were evaluated before surgery and at last follow-up. Lengthening of multi-level soft tissues, tendon transfers and botulinum toxin A injections were documented, as well as the level of FDO.

The FA was assessed with the child in the prone position; the internal (IR) and external rotation (ER) of the hip was measured while not allowing pelvis rotation. Over 70° of IR and limited ER (<30°) was considered to indicate the existence of increased FA. The trochanteric prominence test was also used in patients who had undergone previous hip surgery, and a CT scan was performed to measure the absolute FA. If the gait abnormalities were relevant with increased FA, FDO with multi-level surgery was considered for patient.

Video analysis was used to underpin all operative procedures, which were performed based on analysis of both the clinical findings and video analysis. Proximal FDO (pFDO) was the preferred surgical procedure for children between 5 and 8 years old. In these cases, the patient was in the prone position, and osteotomy was performed at the level of trochanter minor and fixed with a blade plate. Dennis–Brown orthosis was used in combination with a custom-made brace to keep the knees in extension for 4 weeks postoperatively. For children older than 8 years, distal FDO (dFDO) was the preferred surgical approach. In these cases, the patient was in the supine position, and osteotomy was performed at the level of the metaphysis and diaphysis junction and fixed with a dynamic compression plate. A knee immobilizer was applied for 4 weeks postoperatively. Orthotics can be removed during the physical therapies and for hygienic care. The level of osteotomy, type of implant selection, postoperative bracing and/or casting may difference in accordance with the surgical procedure.

The IR was corrected to approximately 30° and the ER to approximately 50° with both proximal and distal de-rotations. Patients were referred back to their rehabilitation centers as soon as possible after surgery to continue muscle exercises and strengthening. Complications were classified as infection, non-union, fracture around the implant and re-increase of FA.

For video-based gait assessment, we used the Edinburgh Visual Gait Score (EVGS) and compared preoperative scores with those at the last follow-up [26]. Three-dimensional gait analysis was assessed using the BTS motion analysis system (Elite Eliclinic, BTS, Milan, Italy) consisting of six cameras and two force plates. A number of the patients enrolled in the study were found to have an appropriate gait, based on analysis before surgery and at a minimum of 2 years after surgery. Data on temporospatial parameters (stance phase  %, swing phase  %, double support time  %, cadence, gait velocity, step length, stride length, step width) and kinematic data (mean pelvic tilt, mean pelvic rotation, peak hip flexion in swing, mean foot progression angle (FPA) in mid-stance, mean hip rotation angle at the end of loading response, maximum knee flexion in swing) were collected, and preoperative values were compared with those at the last follow-up.

This research was approved by the Institutional Review Board of the Department of Orthopedics, Istanbul Faculty of Medicine, Istanbul University. The study complies with the Declaration of Helsinki statement on medical protocol and ethics. All patients enrolled in the study provided oral and written informed consent.

### Statistical analysis

Statistical analysis was carried out using the Student *t* test for parametric data, the Mann–Whitney *U* (Wilcoxon rank test) test for non-parametric data and the chi-square test for categorical data, as appropriate (SPSS v18.0; IBM Corp., Armonk, NY). The Kolmogov–Smirnov and Shapiro–Wilk tests were used for normalization. A *p* value of ≤0.05 was considered to be significant. For those interventions which were done bilaterally, only one side were included in the statistical analysis.

## Results

A total of 93 patients (55 % girls, 45 % boys) with 175 operated extremities (82 % bilateral, 18 % unilateral) included in this study. Based on computerized gait analyses, the gait was appropriate in 18 patients (35 limbs; 20 % of all extremities). Mean age at initial surgery was 6.2 ± 3.1 (standard deviation) years. The average age at the follow-up examination was 6.3 ± 3.7 (range 2–16) years. The most common diagnosis was spastic diplegia (82 patients, 75.2 %), followed by tetraplegia (11 patients, 10.2 %).

Hamstring (67.5 %), adductor (44.8 %) and gastrocnemius (38.6 %) release were the most common soft tissue procedures performed simultaneously with FDO. The soft tissue procedures undertaken are shown in Table 1. pFDO was performed on 84 extremities of 45 patients with a mean age of 6.6 ± 1.3 years. dFDO was performed on 89 extremities of 48 patients with a mean age of 10.7 ± 2.4 years. In addition to femoral osteotomies, three periacetabuler Dega osteotomies were performed.

**Table 1:** List of patients receiving botulinum toxin A injection and undergoing soft tissue surgeries

BTx/A injection and soft tissue surgical procedure	Number of patients (%)
BTx/A injection	15 (16)
Adductor release	41 (44)
Psoas/Iliopsoas release	26 (27)
Hamstring release	56 (67)
Gastrocnemius release	40 (38)
Achilles release	4 (4)
Rectus transfer	8 (9)
Tibialis posterior split transfer	4 (4)

*BTx/A* botulinum toxin A

**Fig. 1:**
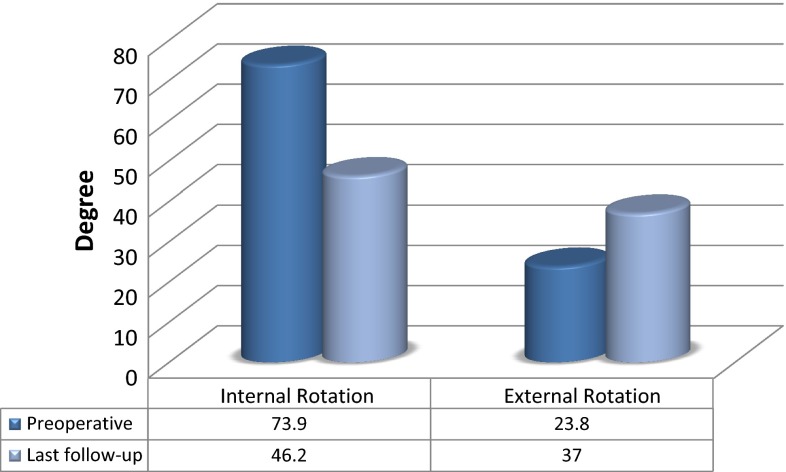
Comparison of preoperative and last follow-up hip rotations. All improvements were statistically significant (*p* < 0.0001)

Comparison of the preoperative values of the parameters under study with the values at last follow-up revealed that the postoperative hip abduction angle had improved (31.3° vs. 34.9°, respectively; *p* < 0.0001), the hip IR had decreased (73.9° vs. 46.2°, respectively; *p* < 0.0001), the hip ER had improved (23.8° vs. 37°, respectively; *p* < 0.0001), Duncan–Ely test positivity had increased (*n* = 26 vs. 52, respectively; *p* < 0.0001) and the popliteal angle had decreased (64.2° vs. 55.8°, respectively; *p* < 0.0001). All changes were statistically significant. A summary of the these clinical findings is given in Table 2 and Fig. 1.

**Table 2:** Clinical findings before surgery and at last follow-up

Clinical parameters studied	Pre-operative	Last follow-up	*p* value
Hip flexion contracture	9.8° ± 4.8°	5.8° ± 3.9°	<0.0001*
Hip abduction	31.3° ± 8.7°	34.9° ± 6.4°	<0.0001*
Hip internal rotation (IR)	73.9° ± 7.8°	46.2° ± 11.3°	<0.0001*
Hip external rotation (ER)	23.8° ± 9.3°	37° ± 8.9°	<0.0001*
Total hip rotation arc	99.2° ± 10°	83.6° ± 10.2°	<0.0001*
Knee flexion contracture	1.03° ± 3.9°	1.3° ± 4.3°	0.506
Popliteal angle	64.2° ± 15.8°	55.8° ± 16.8°	<0.0001*
Thigh–foot angle (±)	45°	48°	0.117
Duncan–Ely test (±)^a^	26	52	<0.0001*

Values are presented as the average ± standard deviation (SD), unless indicated otherwise

* Significant at *p* ≤ 0.05 according to the Wilcoxon test

^a^Number of patients with positive results for the Duncan–Ely test

**Fig. 2:**
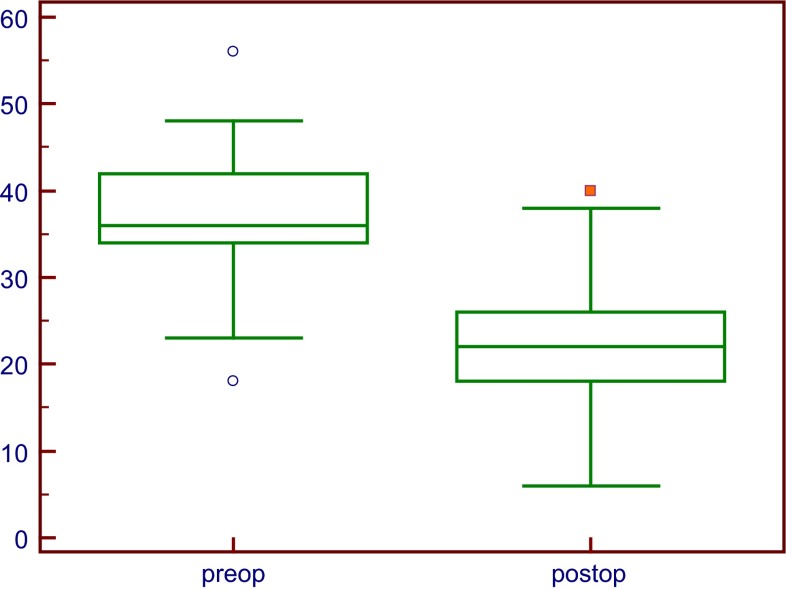
Comparison of total preoperative Edinburgh Visual Gait Scores (EVGS) with total EVGS at last follow-up. The difference was significantly different at *p* < 0.0001

The total EVGS indicated significant improvement after FDO [35.2  ± 6.4 (pre-FDO) vs. 22.5 ± 6.1 (post-FDO); *p* < 0.001] (Fig. 2). Each item/core of the EVGS was also significantly improved at last follow-up (Table 3).

**Table 3:** Significant improvement in total Edinburgh Visual Gait Scores (EVGS) and in all items/cores of the EVGS after derotational osteotomy

EVGS cores/items	Measurement time point		*p* value
	Preoperative	Postoperative	
Foot scores	15.64	9.26	<0.001*
Knee scores	7.27	3.43	<0.001*
Hip scores	3.82	1.46	0.006*
Pelvis scores	3.60	3.02	0.042*
Trunk scores	4.08	3.10	0.004*
Total of all items/cores of EVGS	35.25	22.50	<0.001*
Foot progression angle in stance	1.71	0.34	<0.001*
Knee progression angle in mid-stance	1.64	0.23	<0.001*

* Significant at* p* < 0.05

Computed gait analysis showed significant changes after surgery even in our small group of patients (35 limbs of 18 patients). There was also a significant improvement at the last follow-up in mean FPA in mid-stance [8.1° vs. −16.9° (follow-up); *p* = 0.005] and in mean hip rotation angle at the end of the loading response [−13.9° vs. 5.7° (follow-up); *p* = 0.01]. Maximum hip extension in stance was decreased from 6.7° to 1.5° at last follow-up, but the difference was not statistically significant (*p* = 0.11), and maximum hip flexion in swing was significantly decreased from 42.7° to 35.9° at last follow-up (*p* = 0.032). As the primary aim of FDO was to correct femoral internal rotation in the transverse plane, both maximum hip and knee flexion were improved after surgery (*p* = 0.032 and *p* = 0.052, respectively) in the sagittal plane. The mean pelvic tilt and rotation were similar before and after surgery (Table 4).

**Table 4:** Kinematic parameters

Kinematic parameters	Measurement time point		*p* value
	Pre-operative	Last follow-up	
Mean pelvic tilt	13.6 ± 5.3	11.3 ± 6.5	0.336
Mean pelvic rotation	−0.03 ± 1.4	−0.8 ± 0.8	0.107
Max. hip flexion	49.0 ± 7.4	42.7 ± 9.8	0.032*
Mean hip rotation	−13.9 ± 4.2	5.7 ± 2.8	0.01*
Mean foot progression angle	8.1 ± 12.7	−16.9 ± 10.6	0.005*
Max. knee flexion	62.2 ± 6.7	56.3 ± 7.8	0.052*

Values are presented as the average ± SD

* Significant at* p* < 0.05

At the last follow-up stance time gait cycle (GC)  % was improved [60.2 vs. 65.1 % (follow-up); *p* = 0.02] and swing time GC  % was decreased [39.9 vs. 35.2 % (follow-up); *p* = 0.03]. The amount of single limb stance time was increased from 655 to 813 ms at the last follow-up (*p* = 0.01). Double support time and cadence were both decreased (*p* = 0.032 and *p* = 0.01, respectively). There was a non-significant decrease in gait velocity at the last follow-up (0.88 vs. 0.75; *p* = 0.25). Step length, stride length and step width remained similar at 1 year after FDO (Table 5).

**Table 5:** Temporospatial parameters

Temporospatial parameters	Measurement time point		*p* value
	Pre-operative	Last follow-up	
Stance time (%)	60.2 ± 4.2	65.1 ± 2.3	0.02*
Swing time (%)	39.9 ± 4.2	35.2 ± 3.1	0.03*
Cadence (steps per minute)	122.3 ± 22.3	106.7 ± 16.5	0.01*
Double support time (%)	10.1 ± 3.62	13.5 ± 3.4	0.032*
Step length (mm)	418 ± 107.6	414.3 ± 129.8	0.982
Stride length (mm)	850.7 ± 207	841.7 ± 249.6	0.904
Step width (mm)	166.6 ± 38.5	149.5 ± 51.5	0.410
Gait velocity (m/s)	0.88 ± 0.2	0.75 ± 0.2	0.220

Values are presented as the average ± SD

* Significant at* p* < 0.05

Implant failure was seen in four patients in pFDO patients during the early post-operative period due to poor fixation. Early revision was made with a longer blade plate using a different proximal insertion point. Superficial wound infection was seen in two patients in the dFDO group, which was cured with antibiotic therapy. Non-union was seen in two patients and treated with revision plating and bone graft. Three fractures around the implants required surgical treatment. Recurrence of FA was correlated with younger age and seen in six patients at the last clinic visit. The mean age at FDO was 5.2 ± 0.9 years in patients who had recurrence of increased FA.

## Discussion

Femoral anteversion is a common problem in children with CP and can occur either unilaterally (typically in hemiplegia) or bilaterally (typically in children with bilateral involvement). It is generally assumed that the increased internal femoral torsion is a result of increased muscle tone, most specifically of the medial hamstrings. Excessive increased FA is best corrected with FDO. The aim of this study was to analyze the mid-term benefits of FDO on the clinical outcomes of children with CP, as well as on the temporospatial and kinematic parameters of gait in these children.

Anteversion was measured by physical examination with the child in the prone position. Goniometric measurements of hip IR and ER were made; such measurements have been shown to be reliable and can be used to monitor FA with sufficient accuracy. In the prominence test IR and ER are measured by palpation, but it has been reported that palpation of the greater trochanter can improve the accuracy of this prone examination [10]. However, Chung et al. validated prone hip IR and ER tests, reporting that measuring FA is clinically relevant, reliable (intraclass correlation coefficient = 0.88) and sufficient to evaluate the proximal femoral geometry in the transverse plane in patients with CP.

There are many methods for measuring FA, with each producing slightly different measurements and having some variation in the degree of accuracy. Radiological measurement of FA with a plain X-ray is an obsolete technique and not appropriate if the neck shaft angle is very high (>150°) [27]. Other diagnostic imaging modalities used to measure FA include fluoroscopy, ultrasonography, CT and magnetic resonance [17, 28–32]. Measurements obtained by CT imaging was used in our study. CT imaging is probably the most widely used clinical technique for measuring femoral neck anteversion [31], but in children with severe IR, an absolute measurement of FA is not required prior to surgery [10]. There is also no consensus on when FA needs to be measured accurately. In our study only patients who had undergone prior hip surgery were radiologically examined.

FDO may be performed either at the proximal or distal femur. Among the patients enrolled in our study, pFDO and dFDO were performed in 48 and 52 %, respectively, of all 175 extremities. The complication rate was higher in pFDO patients (12 vs. 5; *p* > 0.05). The most commonly seen complications were early implant failure and re-increase of the FA (6 patients); the mean age of these six patients at surgery was 5.2 ± 0.9 years. As expected, younger patients were found to be prone to recurrence.

In our institution, we prefer the patient to be in the prone position on the operation table during pFDOs as this position allows for an evaluation of hip IR/ER while in hip extension and knee flexion. Many authors advocate that the reason for subluxation in spastic hip is increased FA or internal rotation in addition to the coxa valga [17, 33]. As a beneficial effect of hip centralization and to prevent further luxations, pFDO can be performed in combination with varus osteotomy.

The EVGS is a simple tool for use in video-based gait assessment. It has been validated specifically for use in patients with CP and has a good inter-observer and excellent intra-observer reliability [26]. In our study, video recordings of gait events on the sagittal, coronal and transverse planes were assessed at selected anatomic levels. In this visual analysis, abnormality is severe when the score exceeds “0”. In our series, total visual scores were significantly improved after FDO (36.8 ± 6.3 vs. 22.2 ± 6; *p* < 0.0001), as well as individual ones (Fig. 2; Table 2).

Three-dimensional gait analysis helps confirm the cause and presentation of rotational abnormalities that can be a result of rotational deformities of the femur and/or tibia reflected in abnormal hip rotation and foot progression in the transverse plane. Gait analysis also provides further information about pelvic rotation in the transverse plane, and this information can help the surgeon to interpret transverse plane abnormalities [11]. Akalan et al. studied the gait parameters of CP children with increased FA and compared these with those of children with increased FA who were developing normally [34]. These authors noted that the effects of increased FA differ between a child with CP and a normal child. This observation led them to conclude that before muscle lengthening, FDO should be considered in early stages of growth in CP in order to improve pelvic stability and the knee extensor mechanism [34].

Evaluation of our results revealed significant improvements in the transverse plane kinematics and the time–distance parameters, indicating an improvement in the gait function. Improvements, including those in the mean FPA in mid-stance and mean hip rotation angle at the end of the loading response, were significant at the last follow-up (*p* = 0.005 and* p* = 0.01). Maximum knee flexion (62.2 vs. 56.3; *p* = 0.05) and maximum hip flexion (49 vs. 42.7; *p* = 0.032) had decreased significantly at the last follow-up. Our physical examination and gait analysis results show that the dynamic effects of other procedures, such as hamstring and iliopsoas lengthening, were maintained at the last follow-up and that gait was improved.

As anticipated, in-toeing gait resolved after FDO—and is a second beneficial effect of FDO. The primary purpose of FDO was to correct femoral internal rotation in the transverse plane, with an improvement of in-toeing gait. Both parameters were seen to have improved after surgery in our cohort (*p* = 0.01 and *p* = 0.005, respectively).

Previous studies have shown that the amount of in-toeing can decrease with soft tissue surgeries [35, 36]. Distal hamstring lengthening of contracted medial hamstrings can restrict external rotation of the limb and resolves in-toeing. Also, rectus femoris muscle transfer to the medial hamstrings can increase the external rotation strength of an affected limb [35, 36]. We report severe in-toeing in spastic children, with a mean preoperative hip IR of 73.9° ± 7.7°. The difference between the pre- and postoperative hip IR determined in the clinical examination was 27.7° ± 9.3° (*p* < 0.0001), and the FPA was 25° ± 11.2° (*p* < 0.0001). This significant improvement of in-toeing in children with CP can only be due to the FDO.

Some children with increased FA seem to gain stability by their walking experience over years with a hip IR; thus, a hip IR may provide better stability in stance. Once the deformity is corrected, the children tend to return to the experienced posture of IR [10]. Our study showed that compensating external tibial torsion was more common in those patients who were operated on after reaching an age of 8 years (20 vs. 9; *p* = 0.015), as seen at last follow-up.

There are several limitations to our study which are inherent to its retrospective design and heterogeneous patient characteristics in terms of geographic involvement of the disease. Rather than evaluating clinical examination changes at two different time points (preoperative and last follow-up), it would have been better to include an additional follow-up examination in the relatively short-term at about 1 year postoperative. Evaluating the effect of a single procedure included in a multilevel surgery is always difficult. By controlling other soft tissue procedures and determining the homogeneity of gait abnormalities and functional status of the patients, we may have been able to determine the effects of FDO more precisely. Small changes in kinematic values might have been caused by marker placement, even though this procedure was performed by an experienced researcher. Also, gait analysis combined with surface electromyography and muscle strength measurement by a hand-held dynamometer may provide more accurate results in muscle force analysis. Pre- and postoperative clinical assessments and surgeries were performed by a single experienced pediatric orthopedic surgeon and were therefore reliable.

We reported the mid-term follow-up results of a large number of patients undergoing FDO with multi-level surgery. However, due to an insufficient number of cases with appropriate motion analysis (20 % of all extremities), the gait parameters could not be evaluated accurately. Further study with more gait assessments is required to define the effects of FDO with multi-level surgery.

In conclusion, our data suggest that FDO is a possible treatment option for the correction of FA and associated in-toeing gait in children with CP. Improvements in clinical and kinematic parameters were observed for both conditions after FDO with multi-level soft tissue release. The most prominent effects of FDO were on transverse plane hip rotation and FPA.
